# Hemothorax caused by costal exostosis injuring diaphragm: a case report and literature review

**DOI:** 10.1186/s13019-022-01984-7

**Published:** 2022-09-06

**Authors:** Ruonan Pan, Xiaoqian Lu, Zhijun Wang, Lijun Duan, Dianbo Cao

**Affiliations:** 1 Department of Radiology, the First Hospital of Jilin University, No. 71 of Xinmin Street, Changchun, Jilin, 130021 China; 2 Pediatric Surgery, the First Hospital of Jilin University, Changchun, 130021 China

**Keywords:** Costal osteochondroma, Hemothorax, CT, Treatment

## Abstract

**Background:**

Osteochondromas, also known as exostoses, are the most common benign tumors of bone and can be classified into isolated and multiple osteochondromas. A great majority of osteochondromas is asymptomatic, painless, slow-growing mass, and incidentally found. However, osteochondromas occurring in adolescence or in adult patients can grow in size and become symptomatic as a result of mechanical irritation of the surrounding soft tissues or peripheral nerves, spinal cord compression, or vascular injury.

**Case presentation:**

We present a case of a 13-year-old girl with spontaneous hemothorax, the cause of which was identified by limited thoracotomy with the aid of video-assisted thoracic surgery to be bleeding from a diaphragmatic laceration incurred by a costal exostosis on the left sixth rib. Preoperative chest computed tomography (CT) depicted a bony projection arising from the rib and bloody effusion in the intrathoracic cavity, but was unable to discern the bleeding cause from the lung or the diaphragm. This case will highlight our awareness that costal exostosis possibly results in bloody pleural effusion. Meanwhile, English literatures about solitary costal exostosis associated with hemothorax were searched in PubMed and nineteen case reports were obtained. Combined our present case with available literature, a comprehensive understanding of this rare disease entity will further be strengthened.

**Conclusions:**

Injury to the diaphragm is the primary cause of hemothorax caused by costal osteochondroma, including the present case. Thoracic CT scan can help establish a diagnosis of preoperative diagnosis of costal osteochondroma. Surgical intervention should be considered for those patients with symptomatic osteochondroma of the rib. Combined with our case and literature, prophylactic surgical removal of intrathoracic exostosis should be advocated even in asymptomatic patients with the presentation of an inward bony spiculation.

## Background

Osteochondromas represent the most common bone tumor accounting for 20–50% of all benign osseous tumors [1]. They develop during bone maturation at the metaphysis in the period from early childhood through the late teens, which can be sessile or pedunculated. Osteochondromas may be solitary or multiple. A percentage of 85% of osteochondromas present as solitary lesions, while 15% occur in the context of hereditary multiple exostoses or familial osteochondromatosis, a genetic disorder that is inherited in an autosomal dominant manner [2]. Osteochondromas tend to occur at the metaphyseal region of the long bones of the extremities, but have also been reported to exist in other areas such as the scapula, pelvis, clavicle, rib and vertebra. The key radiological features of osteochondroma are cortical and marrow continuity between the lesion and the parent bone, and a cartilage cap [3]. When these tumors are found within the thoracic skeleton, clinicians face unusual diagnostic and therapeutic dilemmas. Radiologists must be aware of the potential life-threatening secondary complications from rib osteochondromas and the role of CT scan for full diagnostic work-up. Nowadays, the mainstay of conservative treatment is observed with plain radiographs initially and subsequently by clinical examination. Indicators for surgical therapy include pain, complications, cosmetic reasons, increased risk of malignant transformation, and uncertain diagnosis. Here, a costal osteochondroma manifesting as spontaneous hemothorax is reported and treated successfully via limited thoracotomy with the aid of VAT. Meanwhile, similar English literature about hemothorax caused by solitary rib osteochondromas is also reviewed and incorporated into our case so as to better highlight this disease entity.

## Case presentation

**Fig. 1:**
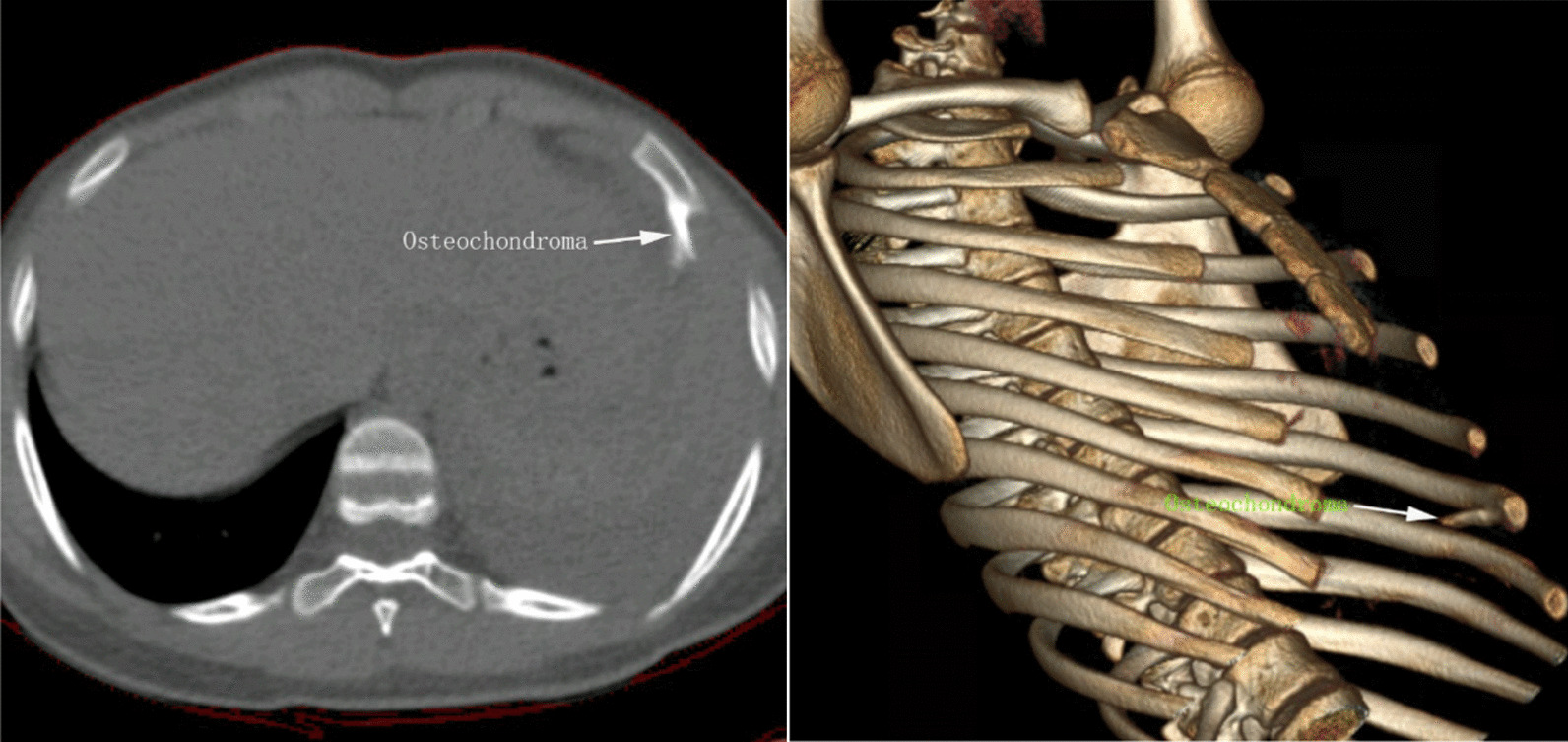
(left): Axial oblique reconstruction showing the inferoposterior course of the osteochondroma. (Right): 3D volume rendered image definitively delineating the orientation of osteochondroma

**Fig. 2:**
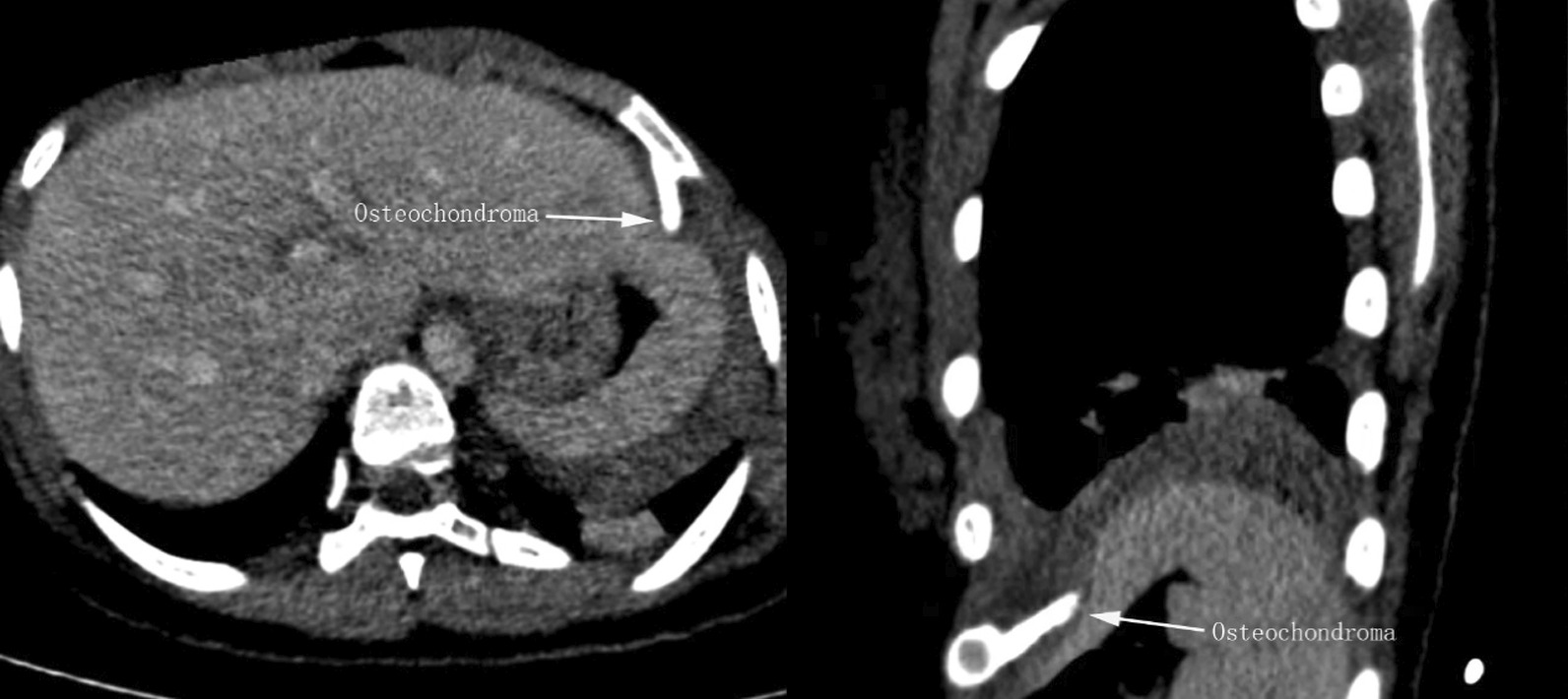
(left), (right): Obliquely axial and sagittal contrast-enhanced CT showing focal impression of hepatic left lobe from the bony lesion, indicative of abutting the diaphragm

On August 12, 2021, a 13-year-old girl presented to our hospital with persistent left shoulder pain for more than 2 months, which was accompanied by left chest pain for 2 days. She denied the history of any familial diseases, recent chest trauma or having anticoagulant drugs. Physical examination revealed reduced respiratory movement and decreased breathing sounds in the left lung field. Percussion sounds of the left chest were solid. Besides, blood hemoglobin levels on admission was 108 g/L(reference range, 110–150 g/L) and clotting parameters were also normal. Chest CT demonstrated a left pleural effusion associated with passive atelectasis of the lung and a 2 cm long spear-shaped osseous proliferation originating from the visceral side of the left sixth rib (Fig. 1). The osseous proliferation showed medullary continuity with the parent rib-bone which prompted the probable diagnosis of osteochondroma. For further diagnosis and treatment of the pleural effusions, the patient underwent ultrasound-guided thoracentesis, and 350 ml of dark blood fluid was aspirated in the intrathoracic cavity, which was indicative of hemothorax. Subsequently, a contrast-enhanced multi-slice computed tomography (MSCT) scan of the thorax ruled out potential pulmonary vascular malformation and neoplasms. The osseous projection abutted the left diaphragm downward (Fig. 2), but there was no visible extravasation of intravenous contrast in the left intrathoracic cavity.

Surprisingly, the patient's hemoglobin level dropped to 88 g/L at the interval of 1 day, a clue of mild anemia from the probability of chronic active bleeding. After homotypic packed red blood cells of 2U were given, the patient was hemodynamically stable. Based on the shape and growth pattern of the bony protrusion on CT and relevant clinical data by now, the hemothorax was probably associated with this costal lesion. Therefore, surgical intervention was necessary to clarify the cause of hemothorax and certain diagnosis of costal lesion.

**Fig. 3:**
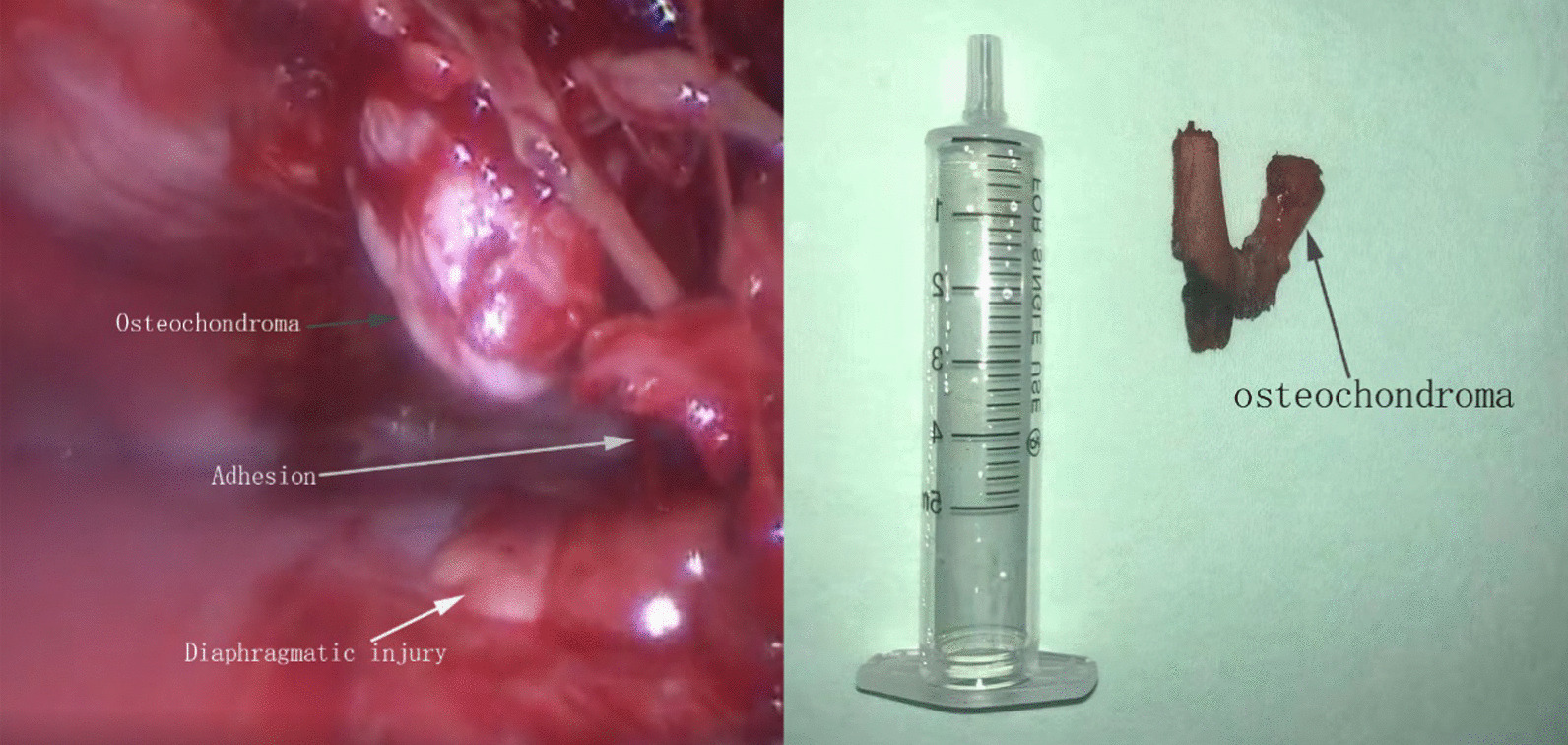
(left): Intraoperative views illustrating the bony lesion compromising the thoracic cavity, diaphragmatic injuries and serious pleural adhesion. (Right): Resected specimen consisted of part of the left sixth rib and an attached lesion of bony protrusion (arrow)

On August 25, 2021, exploratory VAT revealed that a bony protrusion spanned from the medial aspect of the left sixth rib toward the chest cavity, in close contact with the diaphragm. The diaphragm had been lacerated by the bony protrusion (Fig. 3) and was locally covered with residual clotted blood and fibrin layers, suggesting the aetiology of bloody pleural effusion. There was no obvious injury to the visceral pleura, but heavy adhesion of pleural space was noticed. Then the bony protrusion was excised along with partial anterior sixth rib through limited thoracotomy with the aid of VAT. After the closure of wounds, a chest tube was inserted and left until the postoperative 7th day. The gross specimen consisted of rib part and an attached bony protrusion (Fig. 3). Pathology confirms the osseous proliferation consistent with an osteochondromatous proliferation with no signs of dysplasia or concern of malignancy, namely osteochondroma. The patient was discharged home on postoperative 8th day in good clinical condition. At a 6-month follow-up, there was no evidence of any residual or recurrent exostosis.

## Discussion

Osteochondromas, also known as exostoses, are the most common benign tumors of bone and can be classified into isolated and multiple osteochondroma, and they can be observed in 1–2% of the population [4]. The multiple form is an autosomal dominant syndrome referred to as hereditary multiple exostosis or familial osteochondromatosis, accounting for 55% of rib osteochondromas [5]. Osteochondromas are lesions on the surface of the bone composed of both cortical and medullary bone with hyaline cartilage caps, which tend to grow away from the joint through tensile forces of tendons and ligaments. The presence of cortical and medullary continuity of the tumor with the underlying bone is a pathognomonic feature that establishes the diagnosis.

Osteochondromas tend to occur at the metaphyseal region of the long bones of the extremities, but have also been reported to exist in other areas such as the scapula, pelvis, clavicle, rib and vertebra. Among primary chest wall tumors primarily occurring in the rib, fibrous dysplasia is the most common cause of a benign rib growth, followed by osteochondromas [6]. Osteochondromas of the ribs generally originate from the costochondral junction but may also occur at the costovertebral junction. Despite our patient coming in with an osteochondroma slightly more lateral to the costochondral junction, it was histopathologically confirmed to be consistent with a benign osteochondromatous proliferation. A great majority of osteochondromas is asymptomatic, painless, slow-growing mass, and incidentally found. Those symptomatic osteochondromas usually present in younger patients, 75–80% are discovered before the 20th year of age. However, osteochondromas occurring in adolescence or in adult patients can grow in size and become symptomatic as a result of mechanical irritation of the surrounding soft tissues or peripheral nerves, spinal cord compression, or vascular injury. In the case of a patient with a symptomatic osteochondroma of the rib, symptoms may range from hemothorax or pneumothorax to cardiac symptoms, diaphragmatic rupture and spinal nerve injuries due to extrinsic compression, and a palpable lump or malignant transformation. The diagnosis of the patient is osteochondroma located on the rib and manifests as spontaneous hemothorax owing to the diaphragmatic injury. In addition, our patient reported her repeated left shoulder pain lasting for more than 2 months, which was probably a clue of left diaphragm friction by the exostosis. The mechanisms underlying diaphragmatic injury due to exostosis include direct force by a sharp bony spur or repetitive erosion by particularly pointed bony extrusions during respiratory movements. Injury to the diaphragm, pleura, heart, and lung have all been reported rarely [7–21], and they could cause a life-threatening condition if untreated timely. Most previous reports have also not addressed any significant traumatic event or impact prior to the occurrence of symptoms [7–10, 13–18, 21–23], including the case of our patient. Literatures concerning solitary rib osteochondroma leading to hemothorax are listed in the Table 1, which demonstrates different organs injury including the diaphragm, lung, pleura and pericardium in turn. Occasionally it is unable to judge the exact cause of hemothorax, but a favorable outcome after the resection of solitary rib exostosis.

**Table 1:** Cases of spontaneous hemothorax caused by solitary costal exostosis from the literature

Author/year	Age/ sex	History	Induced factors	Symptoms	Diagnostic methods	Radiology findings			Treatment	Injured site
						Costal exostosis	Pleural effusion	Other findings		
R. A. Propper/ 1980 [7]	9/M	Osteochondromas elsewhere, family history of familial multiple exostosis, right shoulder pain	Unknow	Right-sided chest pain	Thoracentesis, CXR	Right 6th	Yes	None	Thoracotomy	Pleura
J R Reynolds /1990 [8]	14/M	Shoulder pain for two weeks	None	Left-sided chest pain, dizziness, dyspnea	Thoracentesis, CXR, CT	Left 7th	Yes	None	Thoracotomy	Diaphragm
S M Tomares /1994 [9]	3/M	Osteochondromas elsewhere	Unknow	Right-sided chest pain	Thoracentesis, CXR, CT	Right 6th	Yes	Atelectasis, pneumonia	VAT	Pleura
N K arrison/1994 [22]	36/F	None	Unknow	Left-sided chest pain, dyspnea	Thoracic drainage, CXR, CT	Left 4th	Yes	None	Thoracotomy	Unknow
David A. Simansky/1997 [10]	17/M	Family history of familial multiple exostosis	None	Dyspnea, syncope	Thoracentesis, CXR, CT	Right 9th	Yes	None	VAT	Diaphragm
Keith G. Buchan /2001 [11]	21/M	None	Strenuous exercise	Left-sided chest pain	Thoracentesis, CXR, CT	Left 4th	Yes	None	Thoracotomy	Pericardium
Waseem M. Hajjar/2003 [12]	20/M	None	Sneeze	Right-sided chest pain, shortness of breath	Thoracic drainage, CXR, CT	Right 6th	Yes	None	Thoracotomy	Diaphragm
Alessandro Bini/2003 [13]	36/M	Spontaneous hydropneumothorax	Unknow	Right-sided chest pain	Thoracic drainage, CXR, CT	Right 9th	Yes	None	VAT and limited thoracotomy	Lung
Mai Linh Pham-Duc/2005 [14]	15/F	Low back pain a month ago	Unknow	Chest pain, dyspnea, vasovagal reaction	Thoracentesis, CXR, CT	Left 8th	Yes	None	VAT	Lung
Wook Jin/2005 [15]	11/F	None	None	Left-sided chest pain, dyspnea	Thoracic drainage, CXR, CT	Left 6th	Yes	Mediastinal shift	Thoracotomy	Diaphragm
Kazuhide Matsushima /2006 [16]	13/Man	Osteochondromas elsewhere, family history of familial multiple exostosis	None	Right-sided chest pain	Thoracic drainage, CXR, CT	Right 9th	Yes	None	VAT	Diaphragm
Hsuan-Rong Huang /2006 [24]	9/F	Osteochondromas elsewhere, family history of familial multiple exostosis	Exercise	Right-sided chest pain	Thoracentesis, CXR, CT	Right 7th	Yes	None	Observation	Unknow
A Martino/2007 [17]	13/F	None	None	Right-sided chest pain	Thoracentesis, CXR, CT	Right 4th	Yes	Atelectasis of adjacent lobe	Thoracotomy	Diaphragm
J Graham/2008 [25]	15/M	Osteochondromas elsewhere	Exercise	Chest pain, productive cough, malaise, syncope	US-guided thoracentesis, CXR, CT	Right 6th	Yes	None	Thoracotomy	Unknow
Y. Matsuno/2009 [18]	3/M	Osteochondromas elsewhere, family history of familial multiple exostosis	Unknow	Left-sided chest pain	CXR, CT	Left 7th	Yes	None	VAT	Pericardium
Tomoyuki Nakano/2009 [19]	15/M	None	Exercise	Chest pain	Thoracentesis, CXR, CT	Right 6th	Yes	None	VAT	Diaphragm
Gregory S. Marlowe/2011 [23]	10/M	Osteochondromas elsewhere, family history of familial multiple exostosis	None	Right upper quadrant abdominal pain	CT-guided thoracentesis, CXR, CT	Right 7th	Yes	Pneumothorax caused by thoracentesis	Observation	Unknow
Mital Patel /2015 [20]	48/M	Spontaneous hemothorax occurred 3 times in 2 years	Exercise	Dyspnea, almost syncope	CT, DSA	Right 5th	Yes	Active extravasation of the right phrenic artery	Thoracotomy after transcatheter embolization	Diaphragm
Pavai Arunachalam/ 2020 [21]	7/M	None	None	Left-sided chest pain, dyspnea	US-guided thoracentesis, CXR, US, CT	Left 7th	Yes	None	VAT and limited thoracotomy	Pleura, lung
Present case	13/F	None	None	Left-sided chest pain, left shoulder pain	US-guided thoracentesis, CXR, CT	Left 6th	Yes	Atelectasis of the left lung	VAT and limited thoracotomy	Diaphragm

M Male; F Female; CXR Chest X-ray; CT Computed Tomography; U: Ultrasound; VAT Video-Assisted Thoracic surgery; (English literatures with insufficient information of patients or unavailable full text was excluded)

Osteochondromas are most often diagnosed depending on radiographic evidence. Radiographs are often diagnostic, and however, cross-sectional imaging may be indicated to assess for complications, assess the cartilage cap or in some challenging cases establish the presence of medullary continuity. CT and magnetic resonance imaging (MRI) may be of some use in defining the extent of tumor spread locally. Compared with plain X-ray film, cross-sectional imaging offers a more accurate tool in the diagnosis of this condition. Recognition of the radiologic spectrum of osteochondroma and its variants usually allows prospective diagnosis and differentiation of the numerous potential complications, thus helping guide therapy and improving patient management [26]. As described in literature review and our patient, currently CT investigation has become the main method. Its reconstructed image and 3D volume rendering clearly described the morphology, extent and growth pattern of solitary osteochondroma.

Surgical removal of osteochondromas is not usually indicated, especially in childhood. However, surgical resection is indicated for osteochondromas developing in adolescence after puberty or in adult patients with pain, increased size, mechanical complications and malignant transformation. Surgical management of thoracic osteochondroma, with excision for painful, symptomatic, malignant lesions or lesions adjudged to be at risk of intrathoracic complications, yields good outcomes in terms of symptom control, establishing histologic diagnosis, and prevention of thoracic complications [27]. Cases of rib exostosis were surgically approached using different techniques. Minimally invasive thoracoscopic techniques are the preferred method for surgical management of patients with symptomatic costal osteochondromas, but mini-incision thoracotomy is needed depending on factors including dimension of the rib lesion and localization involving the rib [9, 10, 13, 14, 16, 18, 19, 21]. In our case, costal osteochondroma was resected safely via limited thoracotomy with the aid of VAT. VAT can help visualize the internal thoracic structures at locations that are hard to reach and inaccessible through other imaging modalities. Various studies have shown that the surgical resection of osteochondroma is necessary to avoid further complications of hemothorax, pneumothorax or intercostal neuralgia. However, osteochondroma of the ribs can even be conserved if not associated with complications and patient does not need to undergo unnecessary surgery [28].

## Conclusions

Osteochondromas are a very common entity, but rarely occur at the ribs. Osteochondroma of the rib may cause non-specific, occasionally serious complications for mechanical frictions or mass effect. Thoracic CT scan can help establish a diagnosis. Surgical intervention should be considered for those patients with symptomatic osteochondroma of the rib. Combined with our case and literature, prophylactic surgical removal of intrathoracic exostosis should be advocated even in asymptomatic patients with the presentation of an inward bony spiculation.

## Data Availability

We declared that materials described in the manuscript, including all relevant raw data, will be freely available to any scientist wishing to use them for non-commercial purposes, without breaching participant confidentiality.

## Abbreviations

CT: Computed tomography
VAT: Video-assisted thoracic surgery
MSCT: Multi-slice computed tomography
MRI: Magnetic resonance imaging
M: Male
F: Female
CXR: Chest X-ray
US: Ultrasound
